# Generation of deep learning based virtual contrast-enhanced CT images from noncontrast CT images for target volume delineation in cervical cancer radiotherapy

**DOI:** 10.3389/fonc.2026.1764560

**Published:** 2026-03-23

**Authors:** Yukai Chen, Hongling Xie, Shichun Wang, Yuanyuan Chen, Yeqiang Tu, Zhixin Bai, Gang Li, Jiahao Wang, Qiu Tang

**Affiliations:** 1 Department of Radiation Oncology, Women’s Hospital, School of Medicine, Zhejiang University, Hangzhou, China; 2 Hangzhou RuiCare Medtech, Hangzhou, China; 3 Tsinghua University School of Software, Beijing, China; 4 Zhejiang Xiaoshan Hospital, Hangzhou, China

**Keywords:** deep learning, dose, radiotherapy, target volume delineation, virtual contrast CT

## Abstract

**Purpose:**

This study sought to develop an nnTransUNet model for synthesizing virtual contrast-enhanced CT from non-contrast CT, and to evaluate its feasibility for target volume delineation in cervical cancer radiotherapy by comparison with conventional non-contrast and contrast-enhanced CT.

**Methods:**

A total of 210 patients with cervical cancer who underwent noncontrast CT and contrast-enhanced CT scans before and after intravenous administration of iodine contrast agent were selected. The “nnTransUNet” network architecture was used to convert noncontrast CT images into virtual contrast-enhanced CT images. Noncontrast, enhanced and virtual contrast-enhanced CT images were designed for cervical cancer radiotherapy, and their image similarity measurements, supervisor image quality evaluations, CT value distributions and dosimetric evaluations were compared.

**Results:**

The virtual contrast-enhanced CT images achieved scores of 0.958, 48.332, 0.997, and 0.976 in terms of mean squared error (MSE), peak signal-to-noise ratio (PSNR), universal quality index (UQI), and structural similarity index (SSIM), respectively. The Dice similarity coefficient (DSC) and Hausdorff distance (HD) for tumor delineation were 0.95 and<7.1 mm. In the subjective evaluation, the virtual contrast-enhanced CT images achieved a score of 4 points in terms of artefacts, noise, image structural integrity and image distortion, which was consistent with the scores for contrast-enhanced CT images. In terms of anatomical structure clarity, the score was slightly lower than that of the contrast-enhanced CT image (3.7 points vs. 4 points). The CT values of the virtual contrast-enhanced CT images were close to those of the contrast-enhanced CT images, and the CT values of the blood vessels and bone marrow were much greater than those of the noncontrast CT images. Compared with that of contrast-enhanced CT, the dose matching between virtual contrast-enhanced CT and noncontrast CT images was closer, and the relative dose difference in the target area was less than 2%. No significant difference in the organs at risk (OARs) dose distribution between the virtual contrast-enhanced CT images and noncontrast CT images.

**Conclusions:**

We developed a deep learning model based on the nnTransUNet architecture for generating virtual contrast-enhanced CT images from non-contrast CT scans, and validated its feasibility in terms of image quality assessment, radiotherapy dose calculation, and target volume delineation for cervical cancer.

## 1 Introduction

Among female cancers, cervical cancer currently ranks fourth in both incidence and mortality rates worldwide (1). Radiotherapy plays a crucial role in the treatment of cervical cancer (2). Target volume delineation is a critical step in radiotherapy, and magnetic resonance imaging (MRI) is generally regarded as the gold standard for target volume delineation in cervical cancer radiotherapy. However, in clinical practice, MRI is not always available in settings such as some primary hospitals or radiotherapy simulation, where computed tomography (CT) remains the primary imaging modality. Compared with noncontrast CT, contrast-enhanced CT provides not only detailed anatomical information to better delineate the boundary between the primary tumor and surrounding structures but also offers additional hemodynamic characteristics for the specific detection and characterization of intravascular, tightly mixed metastatic lymph nodes (3, 4). These features provide significant advantages in lesion detection, differential diagnosis, clinical staging determination, and radiotherapy planning (5, 6). However, the use of iodine-based contrast agents in contrast-enhanced CT may lead to increased side effects. Furthermore, the introduction of high-atomic-number elements (e.g., iodine) during contrast-enhanced CT can result in overestimation of tissue electron density, which may cause significant dosimetric errors in radiation therapy due to elevated CT values (7, 8).

Currently, cervical cancer radiotherapy usually requires two scans for each patient: one noncontrast CT scan (radiotherapy planning) and one contrast-enhanced CT scan (target delineation). However, repeated plain and contrast-enhanced CT scans may expose patients to higher radiation doses and potential dosimetric errors due to image misalignment caused by unavoidable physiological movements. Furthermore, studies have demonstrated that contrast-enhanced CT exerts a significant impact on dose calculations in IMRT (Intensity-Modulated Radiation Therapy) and SBRT (Stereotactic Body Radiation Therapy) (9). In proton beam radiotherapy dose calculations, contrast-enhanced CT has revealed that the deviation between the calculated distal range (induced by the contrast agent) and the range measured in water can be as large as 3.65 cm (10). Therefore, the influence of the contrast agent must be corrected when contrast-enhanced CT is utilized for radiotherapy dose calculation in high-precision radiotherapy.

Deep learning has emerged as a transformative tool for cervical cancer radiotherapy, with prior work demonstrating its utility in auto-segmentation of pelvic organs at risk (OARs) and tumor target volumes (11–14). However, existing deep learning models for virtual contrast-enhancement CT in radiotherapy focus broadly on abdominal/pelvic malignancies without addressing the unique anatomical and clinical challenges of cervical cancer, and the prevalence of contrast allergies in this patient cohort (15–19). Our work fills this gap by developing a ROI-weighted SSIM loss function tailored to cervical cancer’s anatomical priorities and validating it in a cohort of cervical cancer patients undergoing definitive radiotherapy.

We propose a novel deep learning model named “nnTransUNet” for converting noncontrast CT images to virtual contrast-enhanced CT images. This approach would enable patients to obtain two types of images (noncontrast CT and virtual contrast-enhanced CT) from a single noncontrast CT scan, thereby helping to reduce their radiation exposure and mitigate dosimetric errors. nnTransUNet integrates the advantages of both the nnUNet and TransUNet architectures. nnUNet is a highly flexible and automated deep learning framework specifically designed for medical image segmentation tasks. It enhances segmentation performance across diverse medical image datasets through optimization and adaptive processing (20, 21). TransUNet, on the other hand, combines the global attention mechanism of transformer architectures with the local feature capture capabilities of U-Net’s CNNs, demonstrating strong performance in medical image segmentation tasks (22). With the assistance of TransUNet’s powerful feature extraction capabilities, our model effectively extracts high-level semantic information from noncontrast CT images and maps it to the contrast-enhanced CT feature space. We evaluated the feasibility of this approach for utilizing virtual contrast-enhanced CT imaging in target volume delineation for cervical cancer radiotherapy, and compared the image quality variance and dosimetric differences between noncontrast CT, contrast-enhanced CT, and virtual contrast-enhanced CT images. Our method aims to provide a CT-based virtual contrast-enhanced alternative for scenarios where MRI is unavailable, thereby improving the accuracy of radiotherapy target volume delineation.

## 2 Materials and methods

### 2.1 Image acquisition

CT images from 200 patients with cervical cancer who underwent radiotherapy between September 2022 and December 2024, including both noncontrast and enhanced pelvic CT scans, were retrospectively analyzed. An additional 10 patients’ paired CT images—consisting of noncontrast CT and contrast-enhanced CT scans—were acquired from a separate hospital. These paired CT datasets were obtained retrospectively from patients who had a valid clinical reason to undergo both scans. This retrospective study received approval from the institutional review board of hospital and was performed in accordance with the ethical standards laid down in the 1964 Declaration of Helsinki and all subsequent revisions.

The inclusion criteria for patients were as follows: pathological diagnosis of cervical cancer; having undergone both noncontrast CT scans and contrast-enhanced CT scans; and having received radiotherapy. The exclusion criteria are as follows: patients under monitoring; patients who have not given consent. All scans were acquired in the supine position using a Siemens CT scanner, covering the region from the liver to the pelvic cavity. The scan parameters included the following: 120 kV tube voltage, 150–600 mA tube current, 5 mm slice thickness, 0.72×0.72 mm² to 0.97×0.97 mm² spatial resolution, 512×512 matrix, and 40–50 cm field of view (FOV). The contrast agent used was Omnipaque^®^ (GE Healthcare, China) and the concentration of iodine was 300 mg/ml, the administration of 1.3-1.5 ml/kg body weight. Each patient first underwent a noncontrast CT scan. Subsequently, the nurse administered the contrast agent intravenously through the elbow vein using a high-pressure pump, followed by a venous-phase contrast-enhanced CT scan. The interval between the two scans was less than 2 minutes.

### 2.2 Deep learning method

**Figure 1:**
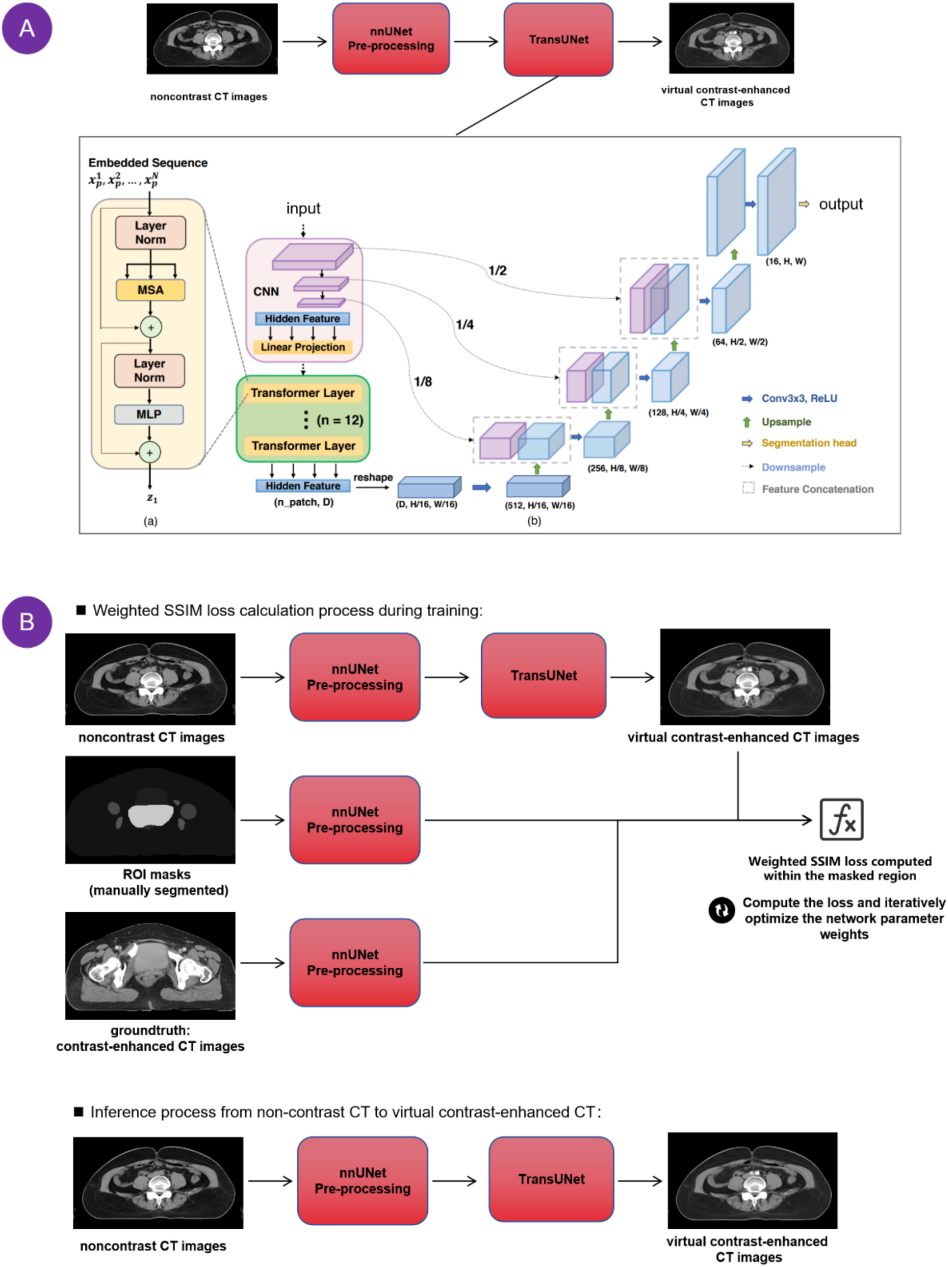
Workflow of the proposed deep learning method. **(A)** Inference process of the proposed method; **(B)** Training process of the proposed method.

We employed the nnTransUNet architecture for our experimental study, detailed nnTransUNet architecture diagram (encoder, transformer bottleneck, decoder, skip connections) as shown in **Figure 1**. The input module receives noncontrast CT images as input. The nnUNet preprocessing component performs intensity normalization and data augmentation on the input images to increase model robustness. The TransUNet encoder then extracts high-level semantic features from the preprocessed images. These features are decoded into virtual contrast-enhanced CT images using a structure similar to that of the TransUNet decoder. The model was trained with a batch size of 32. Train/validation/test split: 180/20/10 (with 5-fold cross validation). Training epochs: 1000, early stopping based on validation loss. Optimizer: AdamW, learning rate 1e-4. Optimal model selection: based on validation ROI weighted SSIM. To optimize and ensure the structural similarity of each region of interest (ROI), we constructed a weighted Structural Similarity Index Measure (SSIM) loss function, as follows in Equation 1:

(1) $${ℒ}_{\text{wSSIM}}=1-\sum_{k\in K}\left(\frac{{\left(A_{k}+\epsilon \right)}^{-1}}{\sum_{j\in K}{\left(A_{j}+\epsilon \right)}^{-1}}\right){\text{SSIM}}_{{\text{Ω}}_{k}}\left(X,Y\right)$$

where κ = {CTV, Rectum, Bladder, Kidney_Left, Kidney_Right, Liver, Body}, $A_{k}=\left|{\text{Ω}}_{k}\right|$ is the area of mask, ϵ>0 is a small constant for numerical stability, ${\text{SSIM}}_{{\text{Ω}}_{k}}\left(X,Y\right)$ denotes the SSIM value computed over region ${\text{Ω}}_{k}$ (using the usual stabilizing constants C1,C2).

The weighted SSIM loss ${ℒ}_{wSSIM}$ is designed to evaluate the structural similarity between the virtual contrast-enhanced CT images and the ground-truth contrast-enhanced CT images in a region-aware manner, focusing specifically on anatomically defined masks such as CTV, Rectum, Bladder, Kidney_Left, Kidney_Right, Liver, and Body. For each region ${\text{Ω}}_{k}$, the Structural Similarity Index (SSIM) is computed by comparing local intensity means, variances, and cross-covariances of the two images within that mask, using the standard SSIM formulation with stabilizing constants to ensure numerical robustness. To integrate these per-region similarity scores into a single metric, the method assigns weights inversely proportional to the area of each mask, thereby amplifying the contribution of smaller yet clinically significant regions (such as the CTV) while attenuating the effect of larger regions (such as the body). These weights are normalized across all masks so that the total contribution sums to one, guaranteeing that the resulting score is interpretable as a convex combination of regional SSIMs. The final loss value is then obtained as one minus the weighted SSIM, making it suitable for minimization during network training. The key advantage of this formulation is that it explicitly balances the influence of anatomical structures of different sizes: small regions, which are often critical for clinical decision-making but would otherwise be dominated by the statistics of larger regions, are given proportionally higher importance. As a result, the loss function not only preserves global image fidelity but also enforces finer structural consistency in small and clinically relevant anatomical regions, which is particularly beneficial in radiotherapy planning and related medical imaging applications.

A 9:1 split ensures the training cohort captures cervical cancer anatomical variability (e.g., tumor size, pelvic anatomy) (23). The dataset was split into 180 training/20 internal test cases (9:1) and 10 external test cases (separate institution) to balance statistical power and real-world validation. For robustness, 5-fold stratified cross-validation was performed on the 180 training cases; 10 external cases tested cross-scanner generalizability, critical for clinical adoption.

### 2.3 Image quality evaluation

Two radiation oncologists delineated the tumor and OARs on three types of images—noncontrast CT, contrast-enhanced CT, and virtual contrast-enhanced CT—based on the patients’ imaging data.

#### 2.3.1 Image similarity measurement

In this experiment, the mean square error (MSE), peak signal-to-noise ratio (PSNR), universal quality index (UQI), and structural similarity (SSIM) were used to quantify and analyze the accuracy of the automatic reconstruction, as shown in Equations 2–5 (24).

(2) $$\text{MSE}=\frac{1}{n}\sum_{i=1}^{n}{\left(x_{i}-y_{i}\right)}^{2}\;$$

In the function above, ${\text{x}}_{\text{i}}$ represents the real data, and ${\text{y}}_{\text{i}}$ represents the fitted data. The value range of the MSE is [0,+∞), and the closer to 0 the value is, the greater the similarity between the two images and the better the model fit. When MSE = 0, the two images are identical (25).

(3) $$\text{PSNR}=10\log_{10}\frac{{\text{m}}^{2}}{\text{MSE}}$$

The MSE represents the mean square error of the two images, and m is the maximum value that can be obtained from the image pixel. The larger the value is, the greater the similarity between the two images and the better the model fit.

(4) $$\text{UQI}=\frac{4\mu_{x}\mu_{y}\sigma_{xy}}{\left(\mu_{x}^{2}+\mu_{y}^{2}\right)\left(\sigma_{x}^{2}+\sigma_{y}^{2}\right)}$$

x and y are the two images being compared. ${\text{μ}}_{\text{x}}$ and ${\text{μ}}_{\text{y}}$ are the means of x and y, respectively. ${\text{σ}}_{\text{x}}$ and ${\text{σ}}_{\text{y}}$ are the standard deviations of x and y, respectively. ${\text{σ}}_{\text{xy}}$ is the covariance of x and y. The value range of the UQI is [0, 1]; the closer to 1 the value is, the greater the similarity between the two images and the better the model fit. When UQI = 1, the two images are identical (26).

(5) $$\text{SSIM}=\frac{\left(2\mu_{x}\mu_{y}+c_{1}\right)\left(\sigma_{xy}+c_{2}\right)}{\left(\mu_{x}^{2}+\mu_{y}^{2}+c_{1}\right)\left(\sigma_{x}^{2}+\sigma_{y}^{2}+c_{2}\right)}$$

Like the UQI function, x and y are the two images being compared. ${\text{μ}}_{\text{x}}$ and ${\text{μ}}_{\text{y}}$ are the means of x and y, respectively. ${\text{σ}}_{\text{x}}$ and ${\text{σ}}_{\text{y}}$ are the standard deviations of x and y, respectively. ${\text{σ}}_{\text{xy}}$ is the covariance of x and y. ${\text{c}}_{1}$ and ${\text{c}}_{2}$ are the two constants used to avoid zero in the denominator. The value range of the SSIM is [0, 1]. The closer to 1 the value is, the greater the similarity between the two images and the better the model fit. When SSIM = 0, the two images are completely different; when SSIM = 1, the two images are identical. Meanwhile, MSE, SSIM, and PSNR were computed within segmented ROIs (tumor and organs at risk), ensuring the evaluation focused on clinically critical regions rather than irrelevant background.

The geometric accuracy of tumor delineation on virtual contrast-enhanced CT images was evaluated using the Dice Similarity Coefficient (DSC) and 95th percentile Hausdorff Distance (95% HD) as shown in Equations 6 and 7 (27). The Dice Similarity Coefficient (DSC) calculates the spatial overlap between two regions as follows:

(6) DSC=2|A∩B|/(|A|+|B|)

where |A| and |B| represent the tumor volumes derived from contrast-enhanced CT images and virtual contrast-enhanced CT images, respectively, and |A ∩ B| denotes the volume (or pixel count) of their intersection. For perfect spatial overlap between the two regions, the Dice Similarity Coefficient (DSC) equals 1. Conversely, in cases of minimal or no overlap, this metric approaches 0.

The Hausdorff Distance (HD) was used to quantify the accuracy of the digitized needle trajectories. To exclude outlier distance values, the 95th percentile Hausdorff Distance (95% HD) was selected; it represents the largest surface-to-surface separation among the closest 95% of surface points. The unit of 95% HD is millimeters (mm), and a smaller 95% HD value indicates more accurate segmentation. The Hausdorff Distance (HD) is calculated as follows:

(7) HD (A, B)=max{h(A, B), h(B, A)}

where *h*(*A*,*B*)=max*a*∈*A* min*b*∈*B* ∥*a*−*b*∥ denotes the directed Hausdorff distance from region A to region B, and *h*(*B*,*A*)=max*b*∈*B* min*a*∈*A* ∥*a*−*b*∥ denotes the directed Hausdorff distance from region B to region A. ∥*a*−*b*∥ represents the Euclidean distance between a point *a* in region A and a point *b* in region B.

In addition, we added comprehensive comparisons with representative generative models (Standard UNet, nnUNet, Pix2pix, CycleGAN, Stable-diffusion 3) as baselines, all models were trained and tested on the same dataset with consistent preprocessing.

#### 2.3.2 Subjective image quality comparison

Two experienced physicians, each with more than 10 years of clinical experience, jointly performed the subjective image assessment. During this evaluation, they specifically examined for hallucinatory artifacts within the range of 5 cm above and below the target volume (a critical region for radiotherapy clinical target volume definition). In cases of disagreement, a consensus was reached through discussion. The evaluation utilized a 4-point scoring system (28). The assessment criteria were as follows:

##### 2.3.2.1 Anatomical structure clarity

O 1 point: Fuzzy anatomical structures, unsuitable for diagnosis.
o 2 points: Unclear anatomical details, failing to meet diagnostic requirements.
o 3 points: Partially poor anatomical visibility but adequate for diagnosis.
o 4 points: Clear anatomical structures that fully meet diagnostic requirements.

##### 2.3.2.2 Artefact presence

o 1 point: Numerous artefacts significantly affect the diagnosis.
o 2 points: Prominent artefacts interfering with surrounding tissue evaluation.
o 3 points: Minimal artefacts present.
o 4 points: No discernible artefacts.

##### 2.3.2.3 Noise level

o 1 point: Severe noise, completely unacceptable.
o 2 points: significant noise but still acceptable.
o 3 points: Moderate noise levels.
o 4 points: Slight or negligible noise.

##### 2.3.2.4 Image structural integrity

o 1 point: Over 80% of layers missing.
o 2 points: Over 50% of layers missing.
o 3 points: Less than 20% of layers are missing.
o 4 points: No missing image layers.

##### 2.3.2.5 mage deformation

o 1 point: Severe deformation, unsuitable for diagnosis.
o 2 points: Moderate deformation affecting the diagnosis.
o 3 points: Minimal deformation, no impact on diagnosis.
o 4 points: No image distortion was observed.

It was assumed that the gold standard noncontrast CT images would achieve the highest score across each category, i.e., a score of 4 points each for anatomical clarity, artefact presence, noise level, structural integrity, and deformation.

#### 2.3.3 CT value distribution

The contours of target and OARs were manually redrawn on each CT dataset with senior oncologists. CT values were linearly compared across cross-sectional images in representative regions of noncontrast, enhanced, and virtual contrast-enhanced CT images. Vascular, muscular, adipose, vertebral, and bone marrow structures were evaluated.

### 2.4 Treatment planning and dosimetric evaluations

The patients received whole pelvic external beam radiation therapy (EBRT) according to our clinical protocol and GEC-ESTRO recommendations. EBRT was administered with a total dose of 45 Gy in 25 fractions using intensity-modulated radiation therapy (IMRT). Additionally, if pathological nodes were present, a simultaneous integrated boost of 61.6 Gy over 28 fractions was employed for treatment (29). All treatments were retrospectively planned using IMRT techniques. Dose calculations were performed using the Monaco treatment planning system (version 6.00.11, Elekta Oncology) with a 6-MV photon beam, a Monte Carlo algorithm, a 3 mm calculation grid, and 3% statistical uncertainty.

To investigate dosimetric differences, we designed and optimized radiotherapy plans for contrast-enhanced CT and virtual contrast-enhanced CT images using identical clinical prescriptions. This process was applied to data from 20 internal patients, with the volume-based plan derived from noncontrast CT images serving as the reference plan. The dose parameters for the PTV and OARs were compared across the three treatment regimens. The evaluated volumetric dose parameters included PGTVnd (D2%, D98%, and D100%), PTV (D2%, D5%, D50%, D95%, D98%, and D100%), and risk organ doses (Dmax in the liver, kidney_left, kidney_right, rectum, bladder, marrow, femoral head_left, femoral head_right, and spinal cord).

**Figure 2:**
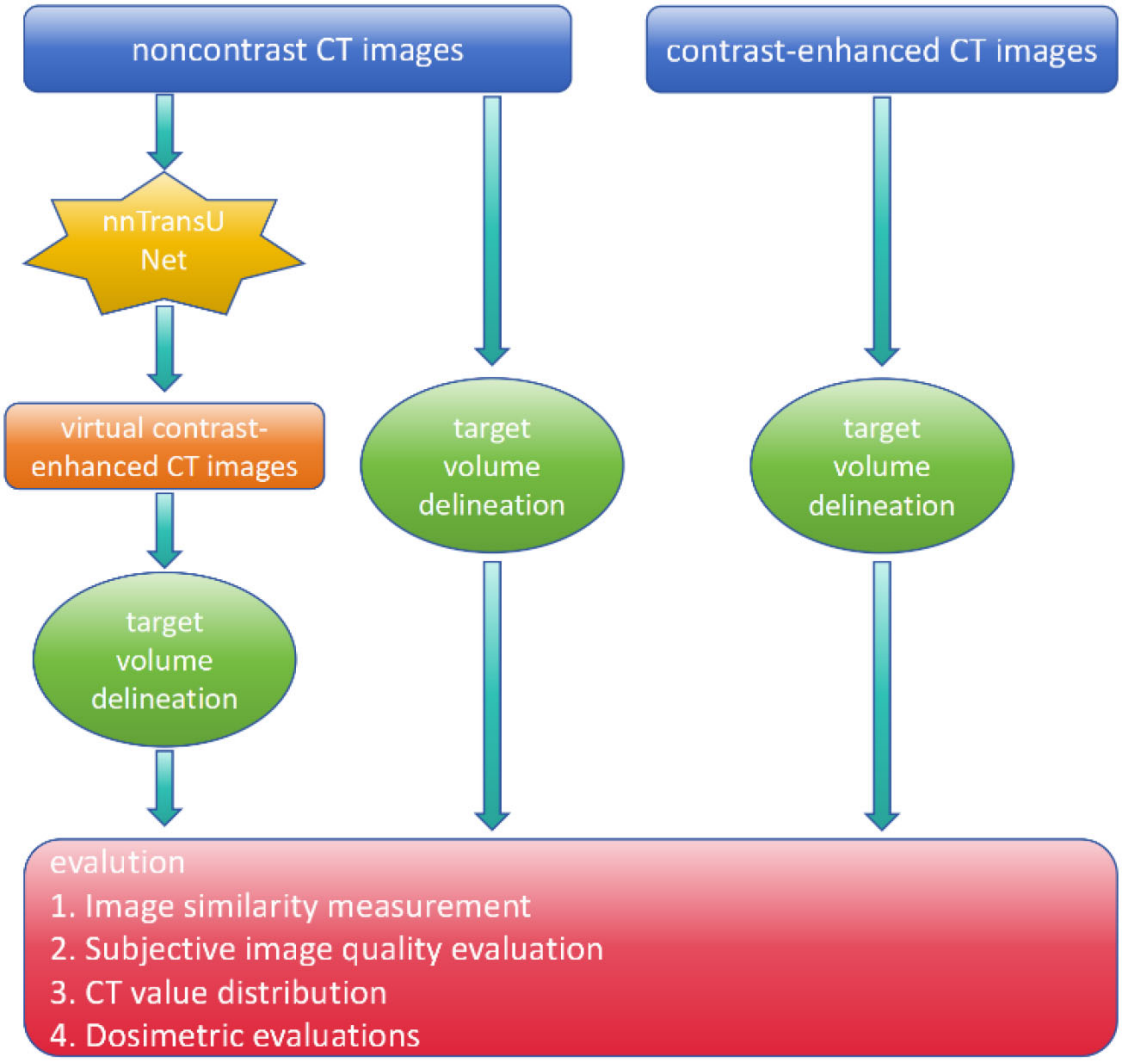
Simplified flowchart of the study pipeline.

The overall workflow of this study is illustrated in **Figure 2**.

### 2.5 Statistical analysis

Statistical analysis was conducted using SPSS 24.0 software (IBM, Armonk, NY, USA). The two-tailed Wilcoxon signed-rank test was applied to assess subjective image quality, image CT values, and dosimetric parameters. P values< 0.05 were considered statistically significant.

## 3 Results

### 3.1 Image similarity measurement

**Figure 3:**
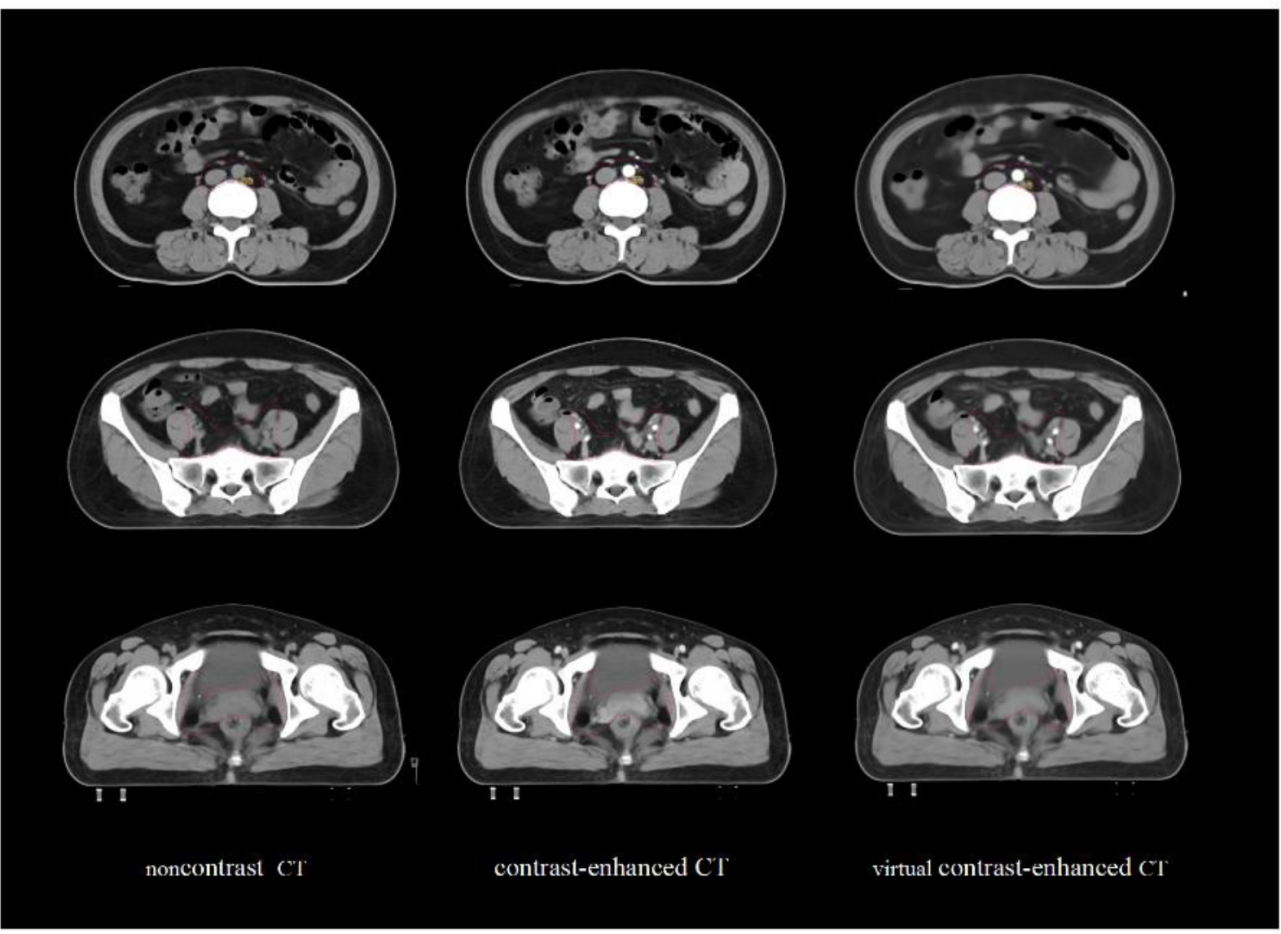
Comparison of noncontrast CT, contrast-enhanced CT and virtual contrast-enhanced CT images in the same slice.

In this experiment, the sample generation outcomes of virtual contrast-enhanced CT images are presented in **Figure 3**. The reconstructed images preserved the structural integrity of the noncontrast CT images while significantly enhancing the iodine contrast agent highlights through the network. **Table 1** presents the quantitative metrics for the automatic reconstruction accuracy of virtual contrast-enhanced CT images, including MSE, PSNR, UQI, and SSIM. These results demonstrate that our model performs well in quantitative evaluation.

**Table 1:** Quantitative results of image quality assessment metrics in the paired real contrast-enhanced CT images against the virtual contrast-enhanced CT images.

Organs at risk	MSE	PSNR	UQI	SSIM
tumor	1.482 ± 0.412	46.982 ± 1.536	0.996 ± 0.002	0.973 ± 0.010
rectum	1.735 ± 0.563	46.104 ± 1.972	0.981 ± 0.006	0.968 ± 0.014
bladder	0.322 ± 0.108	49.876 ± 1.442	0.999 ± 0.001	0.979 ± 0.006
kidney_left	2.864 ± 0.745	44.331 ± 2.115	0.982 ± 0.004	0.961 ± 0.018
kidney_right	2.691 ± 0.803	44.598 ± 2.267	0.983 ± 0.004	0.963 ± 0.017
liver	1.210 ± 0.356	47.215 ± 1.683	0.997 ± 0.002	0.975 ± 0.008
overall	0.958 ± 0.301	48.332 ± 1.142	0.997 ± 0.001	0.976 ± 0.005

The Dice Similarity Coefficient (DSC) for tumor delineation on virtual contrast-enhanced CT images was 0.95, the 95th percentile Hausdorff Distance (95% HD) was 7.1 mm. As shown in **Table 2**, the proposed nnTransUNet architecture outperformed the conventional UNet (with a 7% improvement in GTV DSC) and the standalone nnUNet (with a 4% improvement in GTV DSC), with the most prominent improvements observed in small-volume structures such as lymph nodes (8% improvement over UNet and 5% improvement over nnUNet). The hallucination rate was decreased by 15.1% relative to UNet and by 7.4% relative to nnUNet, which can be directly attributed to the anatomical consistency constraint imposed by the ROI-weighted SSIM loss function.

**Table 2:** Comparison of results between the baseline model and nnTransUNet.

Model	GTV DSC (Mean ± SD)	Lymph node DSC (Mean ± SD)	SSIM (Whole Pelvis)	Hallucination rate (%)
Standard UNet	0.88 ± 0.06	0.79 ± 0.08	0.91 ± 0.05	18.2
nnUNet	0.91 ± 0.04	0.82 ± 0.07	0.95 ± 0.03	10.5
Pix2pix	0.83 ± 0.09	0.74 ± 0.10	0.88 ± 0.07	24.6
CycleGAN	0.85 ± 0.08	0.76 ± 0.09	0.90 ± 0.06	20.8
Stable-diffusion 3	0.87 ± 0.07	0.78 ± 0.08	0.93 ± 0.04	15.9
nnTransUNet (Ours)	0.95 ± 0.03	0.87 ± 0.04	0.97 ± 0.02	3.1

### 3.2 Subjective image quality evaluation

In the comparison of subjective image quality, the scores for artefacts, noise, image structural integrity, and image deformation of the virtual contrast-enhanced CT images were each 4 points, matching those of the contrast-enhanced CT images, with no statistically significant differences (P > 0.05). In terms of anatomical clarity, the score was significantly lower than that of noncontrast CT images (3.70 ± 0.60 vs. 4 points) (P = 0.014), as shown in **Table 3**. No visible hallucinations mismatched with those of the conventional contrast-enhanced CT images were observed.

**Table 3:** Subjective assessment for image quality of virtual contrast-enhanced CT and contrast-enhanced CT.

Images	Clarity of anatomical structure				Artifact				Noise				Image structural integrity				Image deformation			
	1	2	3	4	1	2	3	4	1	2	3	4	1	2	3	4	1	2	3	4
Virtual contrast-enhanced CT	0	2	5	23	0	0	0	30	0	0	0	30	0	0	0	30	0	0	0	30
Contrast-enhanced CT	0	0	0	30	0	0	0	30	0	0	0	30	0	0	0	30	0	0	0	30
P-value	0.014				1				1				1				1			

### 3.3 CT value distribution

**Figure 4:**
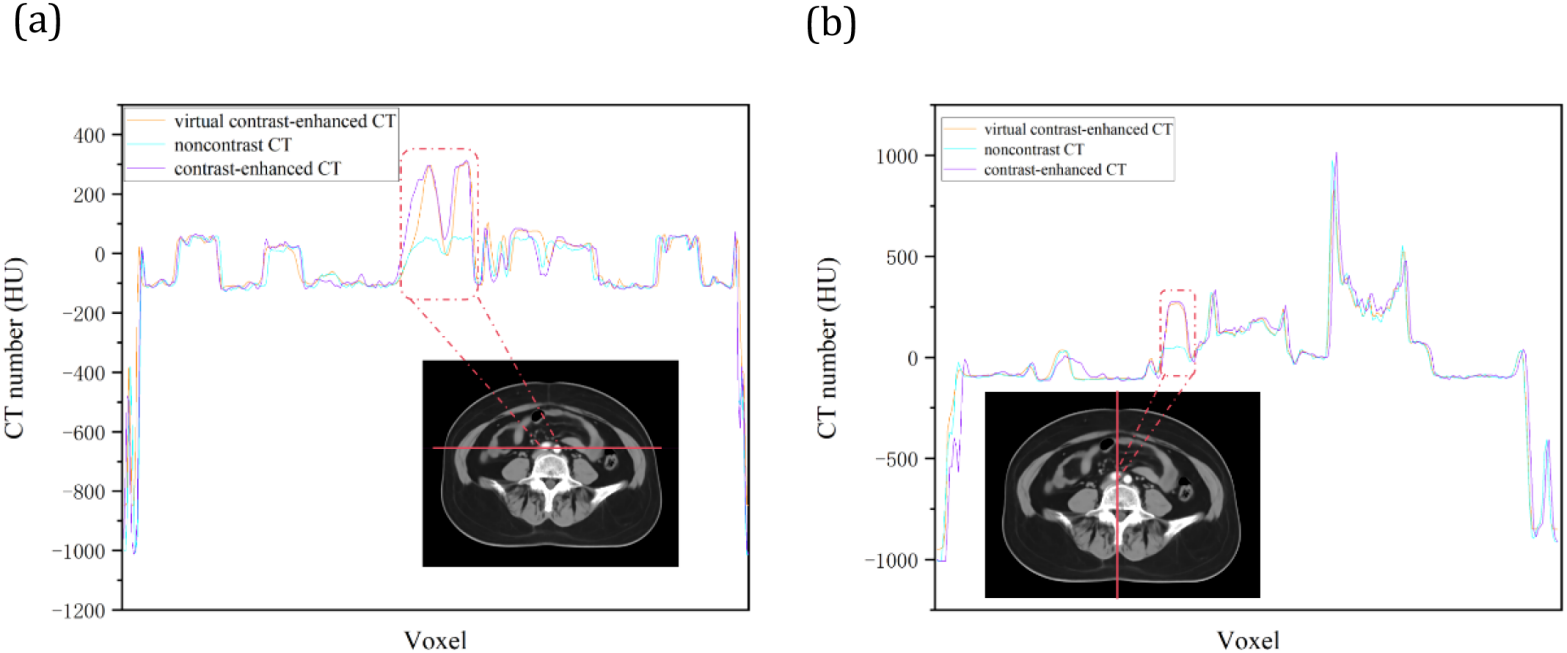
Linear comparison of **(A)** vertical and **(B)** horizontal CT numbers on representative CT image slice.

**Table 4** presents the average CT values for various organs across noncontrast, virtual contrast, and enhanced images. The CT values of vessels and bone marrow in contrast-enhanced CT images were significantly greater than those in noncontrast CT images, with mean differences of 195.8 and 12.86 HU, respectively (p< 0.05). No significant difference was observed between virtual contrast and contrast-enhanced CT images, with mean differences of 14.81 HU for vessels and less than 2 HU each for muscle, fat, vertebrae, and bone marrow. The line profiles of a representative slice are shown in **Figure 4**. Compared with those of noncontrast CT, the CT values of virtual contrast-enhanced CT images are closer to those of contrast-enhanced CT, particularly for vessels.

**Table 4:** Average CT values of different organs in the noncontrast CT, virtual contrast-enhanced CT and contrast-enhanced CT.

Images	Average CT values of organs (HU)				
	Vessels	Muscle	Fat	Vertebrae	Bone marrow
Noncontrast CT	46.67 ± 5.58	54.69 ± 6.39	-104.24 ± 3.60	518.86 ± 164.38	53.01 ± 20.48
Virtual contrast-enhanced CT	242.47 ± 15.64	56.34 ± 4.92	-100.01 ± 6.54	519.69 ± 152.98	65.87 ± 23.04
Contrast-enhanced CT	227.66 ± 29.87	57.34 ± 5.70	-100.28 ± 8.86	519.64 ± 154.69	66.69 ± 24.34

### 3.4 Dosimetric evaluations

**Figure 5:**
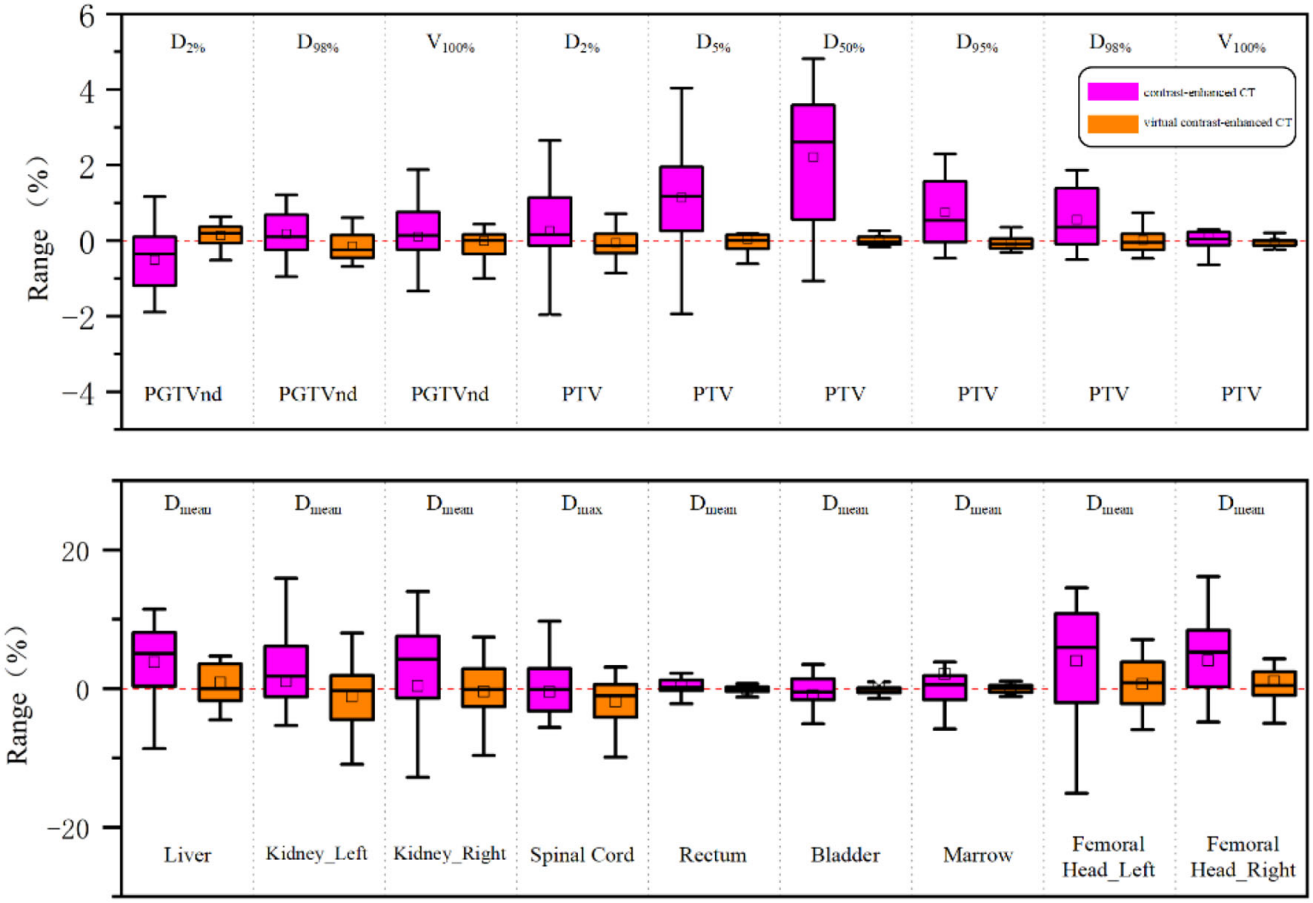
Boxplots of dose differences for the enhanced CT and virtual contrast-enhanced CT plans compared to the reference true noncontrast CT plan.

**Figure 5** shows the dose deviation of enhanced and virtual contrast-enhanced CT images relative to that of noncontrast CT images. Compared with those of contrast-enhanced CT images, virtual contrast-enhanced CT images more closely matched the dose of noncontrast CT images, with a relative dose difference in the target area of less than 1%. The OAR dose–volume parameters for the enhanced, virtual contrast, and noncontrast CT plans are summarized in **Table 5**. There was a statistically significant difference in the mean dose to the bilateral femoral heads between contrast-enhanced CT images (P< 0.05), whereas no significant difference was observed between virtual contrast-enhanced CT and noncontrast CT images.

**Table 5:** Relative dose differences (%) in contrast-enhanced CT and virtual contrast-enhanced CT image compared with in the noncontrast CT image.

Dose parameters in OARs	Contrast-enhanced CT	T	P	Virtual contrast-enhanced CT	T	P
Dmean of Liver	3.83 ± 5.09	1.384	0.183	0.87 ± 5.95	0.970	0.344
Dmean of Kidney_Left	1.06 ± 9.04	-1.497	0.151	-1.12 ± 4.87	-1.247	0.228
Dmean of Kidney_Right	0.41 ± 11.9	-1.273	0.218	-0.43 ± 5.58	-0.535	0.599
Dmax of Spinal Cord	-0.47 ± 5.23	-1.519	0.145	-1.89 ± 3.45	-1.920	0.070
Dmean of Rectum	0.44 ± 1.08	2.182	0.042	-0.05 ± 0.47	-0.463	0.649
Dmean of Bladder	-0.96 ± 4.37	-0.381	0.708	0.61 ± 3.33	0.833	0.415
Dmean of Marrow	2.12 ± 8.33	1.007	0.326	-0.08 ± 0.83	-0.410	0.686
Dmean of Femoral Head_Left	3.40 ± 7.54	3.229	0.004	0.66 ± 3.89	0.951	0.353
Dmean of Femoral Head_Right	4.06 ± 7.15	4.851	<0.001	1.07 ± 4.46	1.078	0.295

## 4 Discussion

Accurate target volume delineation on simulation images is the cornerstone of radiotherapy. One of the current goals in radiotherapy is to ensure image quality while reducing radiation dose, thereby meeting the requirements for clinical diagnosis and treatment evaluation. The remarkable performance of deep learning in medical image processing has unlocked tremendous potential for its practical application in clinical radiotherapy (30). While some studies have described the generation of noncontrast CT images from contrast-enhanced CT images using deep learning techniques, this approach does not benefit patients with allergies to contrast agents (17, 18, 31, 32). This study was designed to provide virtual contrast-enhanced CT images for radiotherapy planning—assisting in the delineation of tumors and OARs—in scenarios where contrast-enhanced CT or MRI is unavailable. In this study, we propose a method based on the nnTransUNet framework to generate virtual contrast-enhanced CT images from noncontrast CT images using a deep learning model. We successfully extended the TransUNet architecture from segmentation tasks to image transformation tasks and further enhanced the model’s performance and robustness by integrating nnUNet’s preprocessing and training strategies. Additionally, we evaluated the generated CT images for radiotherapy target volume delineation and investigated dosimetric differences.

Among cervical cancer patients with contrast agent allergy, standard contrast-enhanced CT for radiotherapy planning cannot be performed. Our model enables generation of virtual contrast-enhanced CT from non-contrast CT in several minutes per case, eliminating the need for alternative staging modalities (e.g., MRI, which has longer scan times and higher costs) in this subset of patients. In low- and middle-income countries, the unavailability of contrast agents and reliance on magnetic resonance imaging are particularly prevalent (33). Our model leverages widely available contrast-enhanced CT to generate virtual contrast-enhanced CT, enabling accurate target volume delineation without contrast agents or advanced imaging. This could expand access to high-quality radiotherapy planning for cervical cancer patients annually in low- and middle-income countries.

The virtual contrast images generated by our proposed model demonstrated high consistency with noncontrast CT images in similarity evaluation metrics. In our study, the model achieved MSE, PSNR, and SSIM values of 0.958, 48.332, and 0.976, respectively, which are comparable to those reported in previous studies (16, 18, 31). We have also included research on converting images from other imaging modalities (such as MRI, PET, CBCT, etc.) into CT images, as detailed in the **Supplementary Table 1**. Liu et al. developed a GAN model capable of directly and automatically synthesizing contrast-enhanced CT images from noncontrast CT images of the abdominal and pelvic regions. Their reported MSE, PSNR, and SSIM values were 226, 19.79, and 0.65, respectively (34). This phenomenon is related to the calculation range of these metrics. Given that all pixel values in the image background are either -1000 or 0, including the background in the calculation will inevitably lead to a reduction in MSE due to the background contribution, while PSNR and SSIM will be inflated by the background (24). In addition, comparisons with the baseline model show that while pure generative models achieve decent global image quality, they suffer from higher hallucination rates and lower anatomical alignment in small ROIs (e.g., pelvic lymph nodes). Our nnTransUNet with weighted ROI level supervision achieves superior structural fidelity and clinical safety, which is essential for cervical cancer radiotherapy. The high DSC (0.95) and low 95% HD (7.1mm) values validate that virtual contrast-enhanced CT images can provide geometrically reliable tumor delineation results, supporting its feasibility as an alternative to in scenarios where MRI or contrast-enhanced CT is unavailable.

In the study by Liu et al., the generated virtual CT images exhibited issues of unnecessary enhancement or insufficient enhancement (34). In our study, we carefully examined the 5-cm range above and below the target volume in our data, and these issues were not observed in our results. This discrepancy may be associated with the characteristics of the datasets: the CT scan dataset collected by Liu et al. includes images from the arterial phase, portal venous phase, and delayed phase. While such data are commonly used in abdominal clinical diagnosis, hemodynamics vary across different anatomical sites, and the enhancement amplitude is strongly correlated with the enhancement phase. Consequently, multiple enhancement phases may hinder the model from accurately identifying the rate of enhancement change in the target region (35, 36). In contrast, our CT scan dataset contains only venous-phase CT images, which are primarily used in radiotherapy. The consistency of the dataset ensures, to a certain extent, the imaging fidelity of the experimental results in this study.

Quantitative CT value analysis revealed that the vascular CT values in contrast-enhanced CT images exhibited the greatest change, which aligns with the findings of Liugang et al (17). Noncontrast CT images are converted into virtual contrast-enhanced CT images via this model. Essentially, by learning the pixel-wise mapping relationship between non-contrast and contrast-enhanced CT images, the model transforms the non-enhanced CT values of substances into their corresponding contrast-enhanced CT values. In the virtual contrast images, while the muscle, fat, vertebral body, and bone marrow CT values remained consistent, the vascular CT values closely matched those of the contrast-enhanced CT images and were significantly greater than those of the noncontrast CT images. This finding indicates that the model has effectively learned the features of high-CT-value substances and iodine contrast agents, demonstrating its fidelity in replicating target-enhanced CT images. The results of the subjective image quality evaluation demonstrate that, compared with contrast-enhanced CT images, virtual contrast-enhanced CT images exhibit excellent performance in terms of artefacts, noise, image structural integrity, and image deformation. Although the scores for anatomical structure clarity were slightly lower, the differences were minimal and did not compromise the conclusions of the study. Image analysis reveals that regions with significant score degradation are mainly concentrated in the liver and kidneys, and this is also reflected in the results of the image quality assessment metrics for these two organs. Given that the model is constructed based on venous-phase contrast-enhanced CT images, whereas the concentration of iodine contrast agent in the liver and kidneys fluctuates across different enhancement phases, we hypothesize that this outcome may arise from the model’s inability to fully capture the temporal variations in iodine contrast agent concentration across phases (37). This limitation could result in incomplete enhancement and alterations in organ density, thereby impairing the accurate characterization of anatomical details.

In this study, we investigated the dosimetric differences in radiotherapy plans derived from virtual contrast-enhanced CT images and real contrast-enhanced CT images, both of which were based on noncontrast CT images. In terms of radiation therapy plan dosimetry, the dose calculation differences between enhanced and virtual contrast-enhanced CT compared with real noncontrast CT were clinically insignificant. Compared with those of contrast-enhanced CT images, the dose parameters from virtual contrast-enhanced CT images were closer to those of real noncontrast CT images. This could be attributed to the fact that virtual contrast-enhanced CT images are generated from noncontrast CT images. Consequently, compared with contrast-enhanced CT images, virtual contrast-enhanced CT images exhibit a smaller potential impact of geometric deviation and a stronger consistency between the target volume and OARs. For PTV mean dose metrics in virtual contrast images, the values were comparable to those of the real noncontrast CT plan, with differences of less than 1%. This consistency aligns with prior studies that used noncontrast CT images to generate virtual contrast-enhanced CT images (16, 17). However, our study revealed up to 2% average differences in OAR doses in virtual comparison images compared with 1% in other studies. This discrepancy arises because we reoptimized the virtual contrast-enhanced CT image plans using the same clinical prescription, whereas other studies copied the irradiation fields to virtual noncontrast CT images without plan optimization. Despite these differences, all OAR metrics remained within acceptable clinical ranges and did not impact our study conclusions.

To our knowledge, this study is the first attempt to use deep learning to transform noncontrast CT images into virtual contrast-enhanced CT images, with the specific aim of assisting radiotherapy target volume delineation. A comprehensive evaluation was conducted across multiple dimensions, including image similarity metrics, quantitative CT values, subjective image assessment, and radiotherapy planning doses. The results demonstrate that this method effectively generates virtual contrast-enhanced CT images from noncontrast CT images, providing reliable tumor delineation results. This innovation facilitates the generation of virtual enhanced images in institutions without contrast-enhanced CT capabilities or in patients where contrast-enhanced CT is contraindicated because of factors such as allergies to contrast media. Furthermore, this approach reduces patients’ radiation exposure and eliminates uncertainty in the registration correlation between enhanced and noncontrast CT images by relying solely on noncontrast CT and deep learning network models.

This study has several limitations. Firstly, this model was trained based on the venous phase data from a specific scanner, and its performance on arterial phase data or data from different manufacturers is not yet clear and requires further research. Secondly, this study is limited by its retrospective design and relatively modest sample size. The limited heterogeneity in patient anatomy, including inadequate representation of postoperative changes, extreme obesity, and other clinical variants, further constrains the generalizability of the present findings. In addition, as a retrospective study, we did not perform quantitative analyses of lesion detection rate, false-positive rate, and false-negative rate, which are crucial for evaluating the risks of image hallucination and lesion omission. This limitation was partly due to the unavailability of PET−CT data in a proportion of patients, which is the reference standard for verifying lesion authenticity and integrity. Future investigations will focus on the development of systematic translational strategies in oncology, including the design of prospective clinical trials with a larger cohort of cervical cancer patients and standardized imaging acquisition protocols, such that all participants undergo PET−CT (the reference standard for lesion validation) in addition to noncontrast CT and contrast-enhanced CT images. This will provide an objective reference for the validation of lesion presence and extent. Further studies will also include cost−effectiveness analyses and integration with magnetic resonance imaging−guided adaptive radiotherapy.

## 5 Conclusion

In this study, we developed an nnTransUNet-based deep learning model for high-quality synthesis of virtual contrast-enhanced CT images from noncontrast CT scans. Through comprehensive multi-parameter comparative analysis, we validated the superior performance of the proposed model in terms of image quality for cervical cancer. Our results demonstrate that the deep learning framework can effectively generate virtual contrast-enhanced CT images from noncontrast CT data, further confirming its feasibility and clinical potential for radiotherapy target volume delineation in cervical cancer.

## Data Availability

The original contributions presented in the study are included in the article/**Supplementary Material**. Further inquiries can be directed to the corresponding author.
