# Supplementary material for: Generation of deep learning based virtual contrast-enhanced CT images from noncontrast CT images for target volume delineation in cervical cancer radiotherapy
Source: Front Oncol. 2026 Mar 23;16:1764560. doi: 10.3389/fonc.2026.1764560 (PMC13051218; doi:10.3389/fonc.2026.1764560)
Supplement: Supplementary file 1 [file Table1.docx]

**Supplemental material 1: Several recent studies on the synthesis of medical CT images**

| **References** | **Dataset** | | **Model** | **Evaluation** | | **Application Remark** |
| --- | --- | --- | --- | --- | --- | --- |
|  | **quantity** | **Modality** | **Architecture** | **Metric** | **Performance** |  |
| Bird et al.^1^ | 90 subjects | Paired MR-CT | cGAN | D95% of PTV | 0.1% (range: −0.5% to 0.7%) | Anorectal CT synthesis |
| Schaefferkoetter et al.^2^ | 60 subjects | 3D MRI, CT, PET images | CycleGAN  patchGAN | PCC | 0.885 | Whole-body CT synthesis |
| Liu et al.^3^ | 164 subjects | MRI, CT | multi-cycle GAN | MAE  ME  PSNR | 0.0416  0.0340  39.1053 | Head-and-neck CT synthesis from MRI |
| Yoo et al.^4^ | 113 pairs | MRI, CT | GAN, RgGAN  CycGAN | PSNR | 36.20 + 0.48  36.09 + 0.44  36.31 + 0.61 | Prostate CT synthesis from MRI |
| Chandrashekar et al.^5^ | 275 subjects | NCCT, CTAs images | CycleGAN  ConGAN | SegAcc  ClasAcc | 86.1%±12.2%  85*.*7% *±* 10*.*4%  93.5% 85.7% | Simulate contrast enhanced CTA images using noncontrast CTs |
| Sun et al.^6^ | 80 subjects | CBCT, CT, MR images | TGAN | PSNR | 30.29 (1.18)  28.19 (1.23)  26.75 (0.97) | Nasopharyngeal CT synthesis |
| Reaungamornrat et al.^7^ | 5461 slices | MRI, CT | RaSGAN | SSIM  PSNR | 0.74, 0.83  26.09, 25.04 | Pelvis from MRI to CT synthesis |
| Li et al.^8^ | 40 subjects | PET, CT images | TCGAN | SSIM  PSNR  VIF  FID | 0.966, 0.930, 0.867  45.66, 1.29, 27.37  0.836, 0.320, 0.342  69.94, 26.11, 55.23 | Brain CT synthesis from PET |

References

1. Bird D, Nix MG, McCallum H, et al. Multicentre, deep learning, synthetic-CT generation for ano-rectal MR-only radiotherapy treatment planning. *Radiotherapy and Oncology*. 2021;156:23-28. doi:10.1016/j.radonc.2020.11.027

2. V J S, D JF, Moraru L. Deep Learning Algorithm for COVID-19 Classification Using Chest X-Ray Images. *Computational and Mathematical Methods in Medicine*. 2021;2021:1-10. doi:10.1155/2021/9269173

3. Liu Y, Chen A, Shi H, et al. CT synthesis from MRI using multi-cycle GAN for head-and-neck radiation therapy. *Computerized Medical Imaging and Graphics*. 2021;91doi:10.1016/j.compmedimag.2021.101953

4. Yoo GS, Luu HM, Kim H, et al. Feasibility of Synthetic Computed Tomography Images Generated from Magnetic Resonance Imaging Scans Using Various Deep Learning Methods in the Planning of Radiation Therapy for Prostate Cancer. *Cancers*. 2021;14(1)doi:10.3390/cancers14010040

5. Chandrashekar A, Handa A, Lapolla P, et al. A Deep Learning Approach to Visualize Aortic Aneurysm Morphology Without the Use of Intravenous Contrast Agents. *Annals of Surgery*. 2023;277(2):e449-e459. doi:10.1097/sla.0000000000004835

6. Sun H, Xi Q, Sun J, et al. Research on new treatment mode of radiotherapy based on pseudo-medical images. *Computer Methods and Programs in Biomedicine*. 2022;221doi:10.1016/j.cmpb.2022.106932

7. Reaungamornrat S, Sari H, Catana C, Kamen A. Multimodal image synthesis based on disentanglement representations of anatomical and modality specific features, learned using uncooperative relativistic GAN. *Medical Image Analysis*. 2022;80doi:10.1016/j.media.2022.102514

8. Li J, Qu Z, Yang Y, Zhang F, Li M, Hu S. TCGAN: a transformer-enhanced GAN for PET synthetic CT. *Biomedical Optics Express*. 2022;13(11)doi:10.1364/boe.467683
